# Hemphagocytic Lymphohistiocytosis Secondary to COVID-19: A Case Report

**DOI:** 10.7759/cureus.19292

**Published:** 2021-11-05

**Authors:** Warda A Naqvi, Muhammad J Bhutta

**Affiliations:** 1 Infectious Diseases, Shifa International Hospital, Islamabad, PAK

**Keywords:** hemophagocytic lymphohistiocytosis (hlh), covid 19, covid-19 in pakistan, post-covid sequelae, covid-19 pneumonia

## Abstract

Hemophagocytic lymphohistiocytosis (HLH) is categorized into primary HLH and secondary HLH. Primary or familial HLH is an autosomal recessive disorder due to mutation in immune regulatory genes. Secondary HLH is an uncommon hyperinflammatory disease triggered by a critical illness (malignancies or viral infection) that induces an uncontrollable excessive immune response, which results in multiorgan failure. Due to the rarity of the syndrome, HLH is associated with worse outcomes. Severe coronavirus disease-19 (COVID-19) is identified as a trigger of HLH, and published literature suggests that patients with severe COVID-19 are at high risk of developing HLH. COVID-19-associated HLH is rarely reported in the literature. Herein we present a case of secondary HLH due to COVID-19 presented in the emergency department with prolonged non-resolving fever.

## Introduction

Hemophagocytic lymphohistiocytosis (HLH) is categorized into primary HLH and secondary HLH. It is mainly reported in the pediatric population but adult cases are increasing. It is a rare disorder affecting one to two people per million. Primary or familial HLH is an autosomal recessive disorder due to mutation in immune regulatory genes. Secondary HLH is an uncommon hyperinflammatory disease triggered by a critical illness (malignancies or viral infection) that induces an uncontrollable excessive immune response, which results in multiorgan failure. Due to the rarity of the syndrome, HLH is associated with worse outcomes. Severe coronavirus disease-19 (COVID-19) is identified as a trigger of HLH, and published literature suggests that patients with severe COVID-19 are at high risk of developing HLH [1]. COVID-19-associated HLH is rarely reported in the literature. Herein we present a case of secondary HLH due to COVID-19 presented in the emergency department with prolonged non-resolving fever.

## Case presentation

A 38-year-old male presented to us with a high-grade fever for the last three months. He also complained of rigors and chills, myalgias, and a productive cough. He had multiple admissions in the past due to a non-resolving fever. His first admission, about three months back, was due to leucopenia and flu-like symptoms. His COVID-19 polymerase chain reaction (PCR) was positive at that time. He also had a history of end-stage renal disease (ESRD) secondary to hypertension for which he ultimately underwent a renal transplant. It was complicated by transplant rejection. Considering his post-transplant status and immunosuppression, he was advised to get admitted to the COVID floor. However, he was discharged on family request. One month later, he was admitted again for myalgia, intermittent fever, cough, and worsening renal failure. He responded to steroids; however, his fever was not resolved despite the use of broad-spectrum antibiotics.

**Figure 1:**
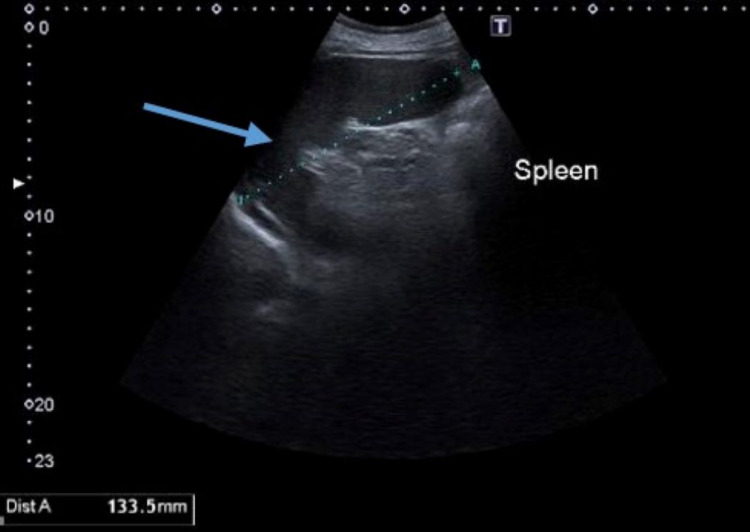
Ultrasound of abdomen Ultrasonography of the patient's abdomen revealed splenomegaly. The blue arrow is pointing towards the spleen. The dotted line shows the entire extent of the spleen.

On examination, he was alert, oriented in time, place, and person. His temperature was 104F, blood pressure 130/70 mmHg, respiratory rate 20 per minute, heart rate 85 per minute, and oxygen saturation of 97 at room temperature. On auscultation, he had bilateral crackles on the base of both lungs. The rest of the examination was unremarkable. High-resolution computed tomography (HRCT) of the lungs revealed persistent heterogeneous ground glass opacities bilaterally in the apex of both lungs. The CT of the patient's abdomen and pelvis did not reveal any abnormalities. Although follow-up abdominal ultrasonography two weeks later revealed splenomegaly (Figure 1).

The patient's blood workup on admission is shown in Table 1.

**Table 1:** Lab parameters RBC: Red blood cell, TLC: Total leukocyte count, ESR: Erythrocyte sedimentation rate

Blood workup	Results	Reference values	Unit
RBC	3.9	4.0-5.1	x E12/L
TLC	4.9	4.0-11.0	x E9/L
Platelet count	201	150-400	x E9/L
Hemoglobin	11.1	14-17	g/dL
ESR	110	<22	
Serum triglyceride	416	<150	mg/dL
Urea nitrogen	13	8.0-20.0	mg/dL
Creatinine	0.8	0.7-1.2	mg/dL
Blood glucose	151	<200	mg/dL
Fibrinogen	502.5	200-400	mg/dL

**Figure 2:**
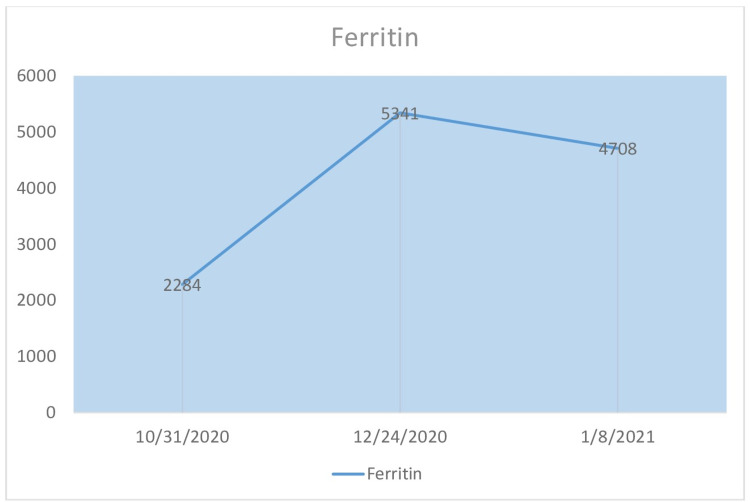
Ferritin The patient's ferritin values over the course of three months.

The patient's ferritin, C-reactive protein (CRP), procalcitonin (PCT), and total leukocyte count (TLC) during his illness are shown in Figure 2 to Figure 5. Liver function tests remained within normal limits throughout the course of his illness.

**Figure 3:**
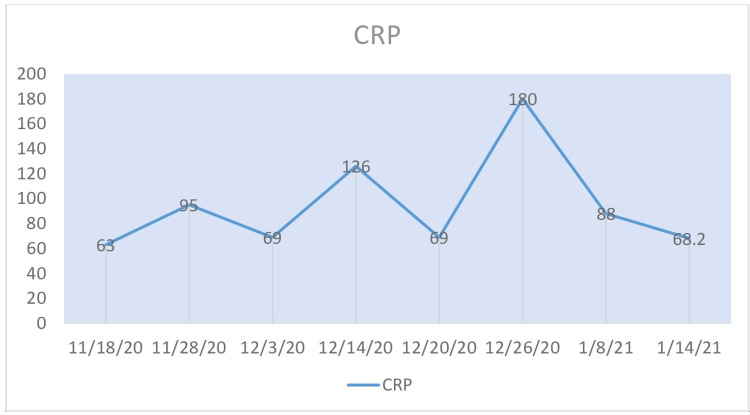
C-reactive protein (CRP) The patient's CRP level over the course of three months.

**Figure 4:**
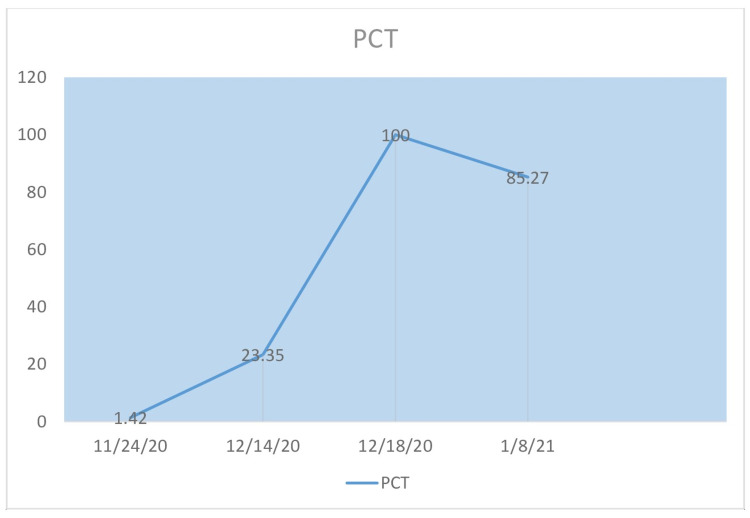
Procalcitonin (PCT) The patient's PCT levels over the course of three months.

**Figure 5:**
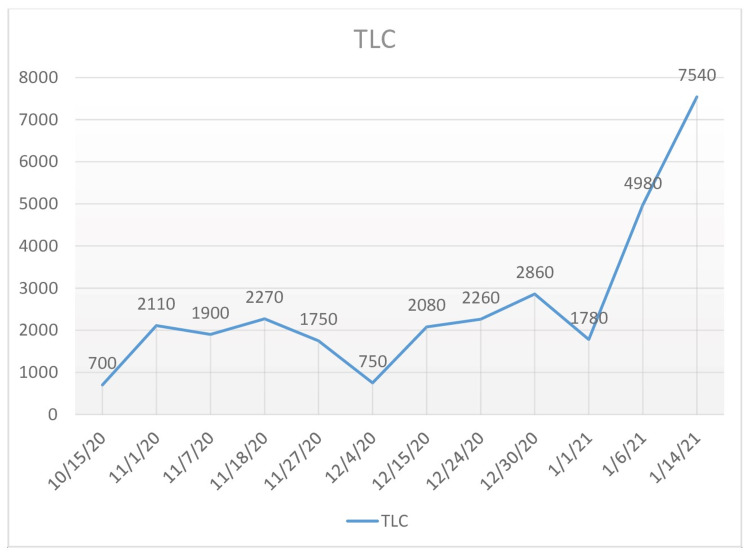
Total Leukocyte Count (TLC) The patient's TLC over the course of three months.

The patient's sputum culture was positive for Pseudomonas Aeruginosa. His blood and urine cultures that were done multiple times during the illness were negative for any organism. He was prescribed carbapenems, linezolid, vancomycin, doxycycline, and voriconazole as an outpatient and inpatient during this course. He was also given trimethoprim/sulfamethoxazole in prophylactic dosage. On admission, he was kept on meropenem, vancomycin, voriconazole, and trimethoprim/sulfamethoxazole. However, his fever was not fully responsive, and his condition worsened.

**Figure 6:**
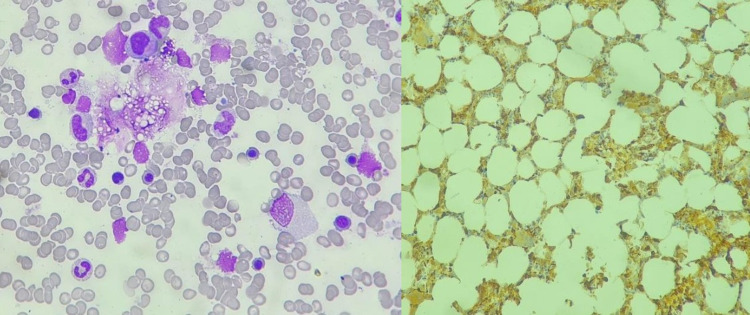
Bone marrow aspirate The patient's bone marrow aspirate shows hypocellular marrow and increased histiocytes with hemophagocytosis.

Further blood workup revealed negative antinuclear antibody (ANA), rheumatoid factor, anti-double-stranded DNA (anti-dsDNA), anti-histone antibodies (ab), anti-nucleosome ab, QuantiFERON, and Mycobacterium tuberculosis (MTB) DNA. He was also negative for human immune deficiency virus (HIV) ab, hepatitis serology, dengue serology, and rapid malarial indirect Coombs test (ICT). A multidisciplinary approach was taken, and the team advised bone marrow aspirate and bronchoscopy. Bronchoscopy was not consented to by the family, and bone marrow aspirate revealed hypocellular marrow, moderately increased histiocytes with hemophagocytosis, and no evidence of malignancy/granuloma (Figure 6). 

Based on the clinical history and morphological findings, HLH secondary to COVID-19 was diagnosed. The patient was given filgrastim 300 mcg subcutaneously (s/c) once daily and recombinant human interleukin-11 3 mcg s/c once daily. He was also started on intravenous dexamethasone 10 mg daily for four days, followed by prednisolone 40 mg daily for the next week, and gradually tapered off. His condition started improving and on his recent follow-up, he is doing well.

## Discussion

COVID-19 induces a wide range of clinical symptoms, ranging from asymptomatic to mild or severe. Severe COVID-19 usually occurs in old age. However, it can also occur in healthy individuals of all ages. One of the factors associated with severe COVID-19 is delayed or impaired T-cell response. Viral infections (most commonly Epstein-Barr virus) are recognized triggers of secondary HLH and may account for almost 35% of cases in adults [2]. A few COVID-19-associated cytokine hyper inflammation reports have been underlined in the literature with only elevated inflammatory cytokines. However, there were no other signs of HLH reported, and the ferritin level was not more than 35000 ug/L [3]. White et al. reported a case of HLH secondary to COVID-19 in the absence of acute respiratory distress syndrome (ARDS) [4]. Tholin et al. also reported a case of HLH due to COVID-19 treated by tocilizumab [5]. Hemophagocytic lymphohistiocytosis is highly uncommon and has a high mortality. The patients usually present critically ill, and the condition worsens rapidly [6].

The primary pathogenesis and physiological abnormality in HLH is cytokine dysfunction, leading to unnecessary accumulation of T-lymphocytes and macrophages (activated histocytes) in many areas of the body. Proliferation, margination, and infiltration of these immune cells into various tissues result in manifestations of HLH [7]. The HLH patients with hypercytokinemia have obstinately raised pro-inflammatory cytokine levels leading to progressive organ failure and death. A triggered immune response could be the mechanism of HLH development in COIVD-19 patients. Severe acute respiratory syndrome coronavirus 2 (SARS-CoV-2) could activate NLR family pyrin domain containing 3 (NLRP3) inflammasome, a potent activator of macrophages, with a significant release of interleukin 1 beta (IL-1b), leading to the release of interleukin-6 (IL-6) subsequently. Molecular docking analysis also predicts the interaction between toll-like receptors-5 and spike glycoprotein of COVID-19, resulting in the release of IL-6. Furthermore, with the downregulation of angiotensin-converting enzyme 2 (ACE 2) receptors in COVID-19, the SARS-CoV-2 could downregulate the anti-proliferative and anti-inflammatory angiotensin (AT) 1-7 pathway [8].

**Figure 7:**
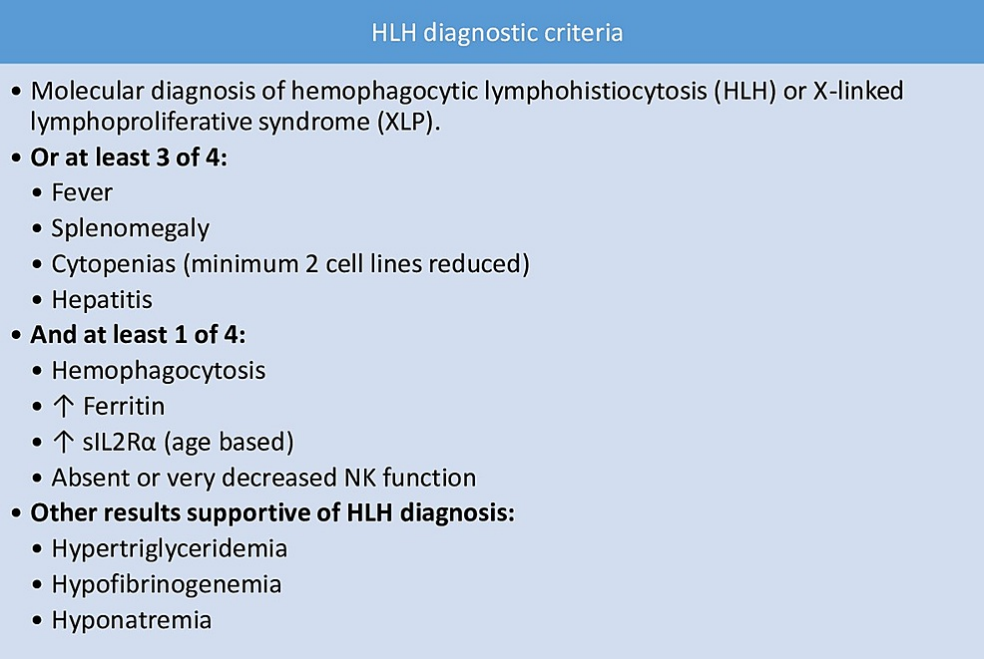
HLH diagnostic criteria sIL2Rα: Soluble interleukin-2 receptor alpha, NK: Natural killer cells, HLH: Hemophagocytic lymphohistiocytosis

Patients with HLH present with febrile illness with the involvement of different systems that mimic other clinical conditions such as viral infections, hepatitis, and encephalitis. Fever and hepatosplenomegaly are identified in many patients with secondary HLH. Our patient had a three-month history of non-resolving fever and splenomegaly on the ultrasound abdomen. The radiologic and clinical picture of the patient suggested severe lung infection. Hemophagocytic lymphohistiocytosis entails a macrophage activation syndrome (MAS) that complicates inflammatory conditions, infectious diseases, and malignancies, making it difficult to diagnose. Hemophagocytic lymphohistiocytosis is diagnosed based on the criteria presented by Bergsten et al. that are listed in Figure 7 [9]. Diagnosis of HLH is made when five of the findings of that criteria are fulfilled.

Treatment of HLH is based on the treatment of the underlying cause. Management involves supportive and immunosuppressive therapy. Therapeutic management involves steroids, etoposide, cyclosporine, and even intrathecal methotrexate in some cases. In a few cases, intravenous immunoglobulin has been used alone or in combination with dexamethasone. Apart from this, anti-interferon-γ monoclonal antibodies and hematopoietic stem cell transplantation are also adopted for treating HLH [10]. The use of interleukin-1 antagonist anakinra has also shown efficacy in secondary HLH in severe COVID-19 patients [11]. Complicated clinical conditions, overdue admission, and multi-organ dysfunction often result in delayed HLH diagnosis.

## Conclusions

Secondary HLH is a potentially fatal condition with high mortality and ought to be considered in the differential diagnosis of patients with prolonged non-resolving fever, and multisystem involvement. Due to its rarity and variable presentation, a high degree of clinical suspicion is warranted to diagnose secondary HLH. A multidisciplinary approach is required in most cases for definitive diagnosis and appropriate management. Early diagnosis and management can prevent long-term morbidity and mortality.
